# Comparative chemical and biological evaluation of *Urtica dioica* extracts obtained by methanol and hexane: antioxidant, cytotoxic, apoptotic, and antimicrobial potentials

**DOI:** 10.1186/s12906-025-05211-3

**Published:** 2025-12-07

**Authors:** Yılmaz Uğur, İrem Nur Menevşe, Muhammed Dündar, Hüseyin Karci, Rukiye Zengin, Abdussamat Güzel

**Affiliations:** 1 Health Services Vocational School, Inonu University, Malatya, 44280 Turkey; 2 Department of Molecular Biology and Genetics, Inonu University, Faculty of Arts and Sciences, Malatya, 44280 Turkey; 3 Republic of Türkiye Ministry of Agriculture and Forestry, Apricot Research Institute, Malatya, 44090 Turkey

**Keywords:** Antimicrobial activity, Antioxidant activity, Apoptosis, Cell cycle, Cytotoxicity, *Urtica dioica*, Solvent polarity

## Abstract

**Background:**

*Urtica dioica* L. is a widely utilized medicinal plant with potential antioxidant, cytotoxic, and antimicrobial properties.

**Methods:**

This study aimed to investigate its multi-target biological activities across four cell lines (A549, MDA-MB-231, HCT116, and BEAS-2B) while evaluating the impact of two extraction solvents (acidified methanol and hexane) on activity outcomes.

**Results:**

The acidified methanolic extract exhibited higher total phenolic (61.25 ± 3.07 mg GAE/g) and flavonoid (51.20 ± 2.01 mg CE/g) content, correlating with superior antioxidant activity (DPPH: 84.36 ± 1.50 mg TE/g; CUPRAC: 174.04 ± 9.54 mg TE/g). In contrast, the hexane extract demonstrated stronger cytotoxicity across cancer cell lines (IC_50_: 3.10–4.12 µg/mL), along with significant induction of apoptosis and G0/G1 cell cycle arrest, despite lower antioxidant capacity. In addition, both extracts increased the protein levels of p21 and cleaved caspase-3, suggesting involvement of cell cycle inhibition and activation of intrinsic apoptotic signalling pathways. Moderate antimicrobial activity was also observed, with inhibition zones ranging from 7 to 10 mm across bacterial and fungal strains.

**Conclusions:**

These findings highlight the bioactive potential of *U. dioica* and the critical role of extraction solvent in modulating its total phenolic and flavonoid contents and biological effects. The observed upregulation of p21 and cleaved caspase-3 further supports the notion that *U. dioica* extracts may exert antiproliferative activity through p21-mediated cell cycle control and caspase-dependent apoptosis. Further in vivo studies and mechanistic investigations are needed to confirm these observations and clarify their potential therapeutic relevance.

**Supplementary Information:**

The online version contains supplementary material available at 10.1186/s12906-025-05211-3.

## Background

Plant-derived natural compounds are considered promising therapeutic agents due to their diverse pharmacological properties, biocompatibility, and relatively low toxicity [1, 2]. Among them, *Urtica dioica* L. (stinging nettle) is a widely distributed and traditionally used medicinal plant in Europe, Asia, and North Africa for conditions such as rheumatism, urinary tract infections, diabetes, hypertension, and skin disorders [3, 4]. Its therapeutic potential is mainly attributed to its rich phytochemical content, including flavonoids, phenolic acids, carotenoids, sterols, vitamins (A, C, K, B-complex), and essential minerals like iron, calcium, magnesium, and potassium [5, 6]. Several studies have confirmed its antioxidant, anti-inflammatory, antimicrobial, hepatoprotective, antidiabetic, and anticancer activities [7].

The efficiency and activity of extracted phytochemicals are significantly influenced by the polarity of the solvent used. Polar solvents like methanol and ethanol are effective for extracting hydrophilic compounds such as phenolics and flavonoids, while non-polar solvents like hexane are better suited for lipophilic substances like terpenoids, sterols, and fatty acids [8]. Therefore, comparative studies using solvents with different polarities are valuable for optimizing both phytochemical yield and biological activity. Although many studies have examined the phytochemical profile and specific biological effects of *U. dioica*, most focused on polar solvents and evaluated single parameters such as antioxidant or antimicrobial activity. Some have explored both polar and non-polar extractions, mainly in the context of antimicrobial properties [9–12]. However, comprehensive studies using acidified polar solvents—such as HCl-acidified methanol—remain limited. These solvents can enhance the hydrolysis of glycosidic bonds, increasing the release of bound phenolics from plant tissues, which in turn boosts the total phenolic content and enhances antioxidant and cytotoxic effects [13, 14]. Beyond enhancing phenolic compound release, acidified methanol extraction may also influence the biological activity of plant extracts by altering the solubility and availability of bioactive compounds capable of modulating membrane integrity and redox signaling in cancer cells. Acidified solvents can hydrolyze glycosidic bonds, potentially liberating aglycone forms of flavonoids, which more readily penetrate cellular membranes and interact with intracellular targets such as mitochondria. These aglycones are generally noted to be more effective in generating or modulating reactive oxygen species (ROS), disrupting mitochondrial membrane potential, and triggering apoptosis through intrinsic pathways [15, 16]. Moreover, to the best of our knowledge, there are limited studies directly comparing the effects of polarity-distinct extracts on cancer and normal cell lines in conjunction with mechanistic endpoints such as apoptosis and cell cycle arrest. This highlights the need for integrative assessments that simultaneously evaluate phenolic content, antioxidant capacity, cytotoxicity, and antimicrobial potential within the same experimental framework.

While the individual antioxidant or cytotoxic properties of *U. dioica* have been widely reported, few studies have systematically compared polar vs. non-polar extracts using a comprehensive panel of assays in a unified experimental setup. In particular, the use of acidified methanol for phenolic enrichment is an underexplored yet promising strategy to improve extract potency, especially regarding antioxidant and antiproliferative effects. Furthermore, this study is among the few to employ multi-cell line screening, including both cancer and non-cancer human cells, along with flow cytometric analysis of apoptosis and cell cycle progression.

In this context, the present study aims to provide an integrative evaluation of *U. dioica* extracts prepared using hexane (non-polar) and methanol acidified with 0.05% HCl (polar) via maceration. The extracts were assessed for the total phenolic content (TPC), total flavonoid content (TFC), and antioxidant activity using the 2,2-diphenyl-1-picrylhydrazyl (DPPH) and the cupric ion reducing antioxidant capacity (CUPRAC) assays to quantify their radical scavenging and reducing capacities. Furthermore, their cytotoxic effects were tested against four human cell lines, including three cancer lines (human breast cancer (MDA-MB-231), human lung cancer (A549), and human colon cancer (HCT116)) and one healthy epithelial line (BEAS-2B), using the 3-(4,5-dimethylthiazol-2-yl)−2,5-diphenyltetrazolium bromide (MTT) assays. To elucidate the underlying mechanisms of cytotoxicity, apoptosis and cell cycle progression were evaluated via flow cytometry.

Additionally, the antimicrobial activities of the extracts were assessed against clinically relevant Gram-positive, Gram-negative, and fungal strains using the disc diffusion method. This comprehensive bioactivity profiling seeks to elucidate the influence of solvent polarity on the chemical and biological properties of *U. dioica*, and to explore its potential as a multifunctional therapeutic agent with anticancer and antimicrobial applications.

## Materials and methods

### Plant material

In this study, *U. dioica* L. subsp. dioica (commonly known as stinging nettle) was collected from private land owned by one of the authors, Abdussamat Güzel, located in the Çelikhan district of Adıyaman province, Türkiye. Permission for plant collection was granted by the landowner. Plant authentication was performed by Dr. Turgay Kolaç (Pharmacognosist, Department of Pharmacy Services, Inonu University), and a voucher specimen was deposited in the Herbarium of Faculty of Pharmacy, Inonu University, under the voucher number TK1377.

The plant material was thoroughly washed with distilled water to remove debris and potential contaminants, and subsequently dried under shade at ambient room temperature to preserve its phytochemical integrity. Once fully dried, the aerial parts of the plant were ground into a fine powder using a laboratory mill. The powdered samples were stored in airtight containers under cool and dry conditions, protected from light, until further analysis.

### Extraction procedure

The powdered aerial parts of *U. dioica* were extracted using two different solvent systems: hexane (non-polar) and methanol acidified with 0.05% HCl (polar). For each extraction, 10 g of plant powder was macerated with 100 mL of the respective solvent at room temperature in the dark for 72 h. This procedure was repeated three times for each solvent, and the filtrates obtained from each cycle were pooled. The combined extracts were filtered through Whatman No. 1 filter paper, and the solvents were evaporated under reduced pressure using a rotary evaporator at 40 °C to obtain the crude dry extracts.

Extraction yields were calculated based on the dry weight of *U. dioica* aerial parts. The methanolic extraction yielded 0.625 g of dry extract from 10 g of powdered plant material, corresponding to an extraction efficiency of 6.25%. The hexane extraction yielded 0.235 g, corresponding to an extraction efficiency of 2.35%.

The dried extracts were stored at 4 °C in amber glass containers protected from light until further analysis. Prior to biological and chemical assays, stock solutions were freshly prepared by dissolving each extract in a suitable solvent— dimethyl sulfoxide (DMSO) for the hexane extract (HE), and a 50:50 (v/v) ethanol:water mixture for the acidified methanolic extract (ME).

### Determination of total phenolic content (TPC)

The TPC of the extracts was determined using the Folin–Ciocalteu reagent method as previously described, with minor modifications [17]. Briefly, 0.5 mL of extract solution was mixed with 2.5 mL of 10% Folin–Ciocalteu reagent. After 5 min of incubation at room temperature, 2 mL of 7.5% sodium carbonate (Na_2_CO_3_) solution was added to the mixture. The final solution was incubated in the dark at room temperature for 30 min. Absorbance was measured at 765 nm using a UV–Vis spectrophotometer (Shimadzu 2000S Model, Japan). A calibration curve was constructed using gallic acid, and the results were expressed as milligrams of gallic acid equivalent per gram of dry extract (mg GAE/g).

### Determination of total flavonoid content (TFC)

The TFC of the plant extracts was determined using the aluminum chloride colorimetric method [18]. In this assay, 0.5 mL of extract solution was mixed with 1.5 mL of methanol, 0.1 mL of 10% aluminum chloride (AlCl_3_) solution, 0.1 mL of 1 M potassium acetate (CH_3_COOK), and 2.8 mL of distilled water. The resulting mixture was incubated at room temperature in the dark for 30 min. After incubation, the absorbance was measured at 415 nm using a UV–Vis spectrophotometer (Shimadzu 2000S Model, Japan). A calibration curve was prepared using catechin as the standard, and results were expressed as milligrams of catechin equivalent per gram of dry extract (mg CE/g).

### Determination of antioxidant capacity

#### DPPH radical scavenging activity

The antioxidant activity of the extracts was evaluated based on their ability to scavenge the stable free radical DPPH. In the assay, 100 µL of extract was added to 3.9 mL of freshly prepared DPPH solution. The mixture was vortexed and incubated in the dark at room temperature for 30 min. The absorbance was then measured at 517 nm using a UV–Vis spectrophotometer (Shimadzu 2000S Model, Japan). Antioxidant capacity was quantified by comparison with a Trolox standard curve and expressed as milligrams of Trolox equivalent per gram of dry extract (mg TE/g) [19].

#### CUPRAC assay

CUPRAC assay was conducted following the method described by Apak et al. [20] with adaptations. In this method, 1 mL of copper(II) chloride solution (10^–2^ M), 1 mL of neocuproine solution (7.5 × 10^–3^ M), and 1 mL of ammonium acetate buffer (pH 7.0) were mixed in a test tube. Subsequently, 100 µL of extract solution was added, and the final volume was brought to 4.1 mL with distilled water. The mixture was incubated in the dark at room temperature for 1 h. Absorbance was recorded at 450 nm using a UV–Vis spectrophotometer (Shimadzu 2000S Model, Japan). The antioxidant capacity was calculated based on a Trolox standard curve and reported as milligrams of Trolox equivalent per gram of dry extract (mg TE/g).

#### MTT test

Anticancer properties of the samples were reported by Sharma et al. [21], it was evaluated against MDA-MB-231, A549, HCT116, and BEAS-2B cell lines. All cells were cultured in DMEM medium supplemented with 10% fetal bovine serum and 1% penicillin and streptomycin at 37 °C in a 5% CO_2_ atmosphere. After the cells covered the surface of the flask by 70–80%, the old medium was discarded and washed several times with sterile PBS (pH 7.4). Trypsin was then added and distributed evenly on the cell surfaces. After incubation with trypsin for 5 min at 37 °C, trypsin activity was inhibited by adding a twofold volume of fresh medium. The resulting solution was centrifuged at 1000 rpm for 7 min, and then the old medium was replaced with 5 mL of fresh medium. Cells were counted and diluted to obtain a final concentration of 1 × 10^5^ cells/mL, then the cell solution was added to 96-well cell plate wells (1 × 10^4^ cells/well). Plates containing cells were incubated at 37 °C in a 5% CO_2_ atmosphere for 24 h for cell attachment, and anticancer activity analyses of the extracts were performed using the MTT method. For analysis, the test substance was diluted with fresh broth medium to obtain the desired concentration (0.78–200 µg/mL) from the stock while the cells were incubated. The old medium was aspirated from the wells containing the cells and 100 µL of broth containing the test substance was added to the wells. The plates were then incubated at 37 °C in 5% CO_2_ for 24 h. The final concentration of DMSO or ethanol used as solvents did not exceed 0.1% (v/v) in any well. To ensure solvent-related effects were excluded, corresponding vehicle control groups (treated with solvent only) were included in all assays.

After this period, the medium containing the test substance was aspirated from the wells and 10 µL of MTT solution (5 mg/mL) and 90 µL of fresh broth medium were added to each well to obtain a final concentration of 0.5 mg/mL MTT and then incubated for 4 h at 37 °C. Optical density was read at 570 nm and 650 nm in the ELISA reader (Epoch, Biotek, USA). Cell viability percentages were determined using the formula [(570 nm-650 nm) _(test (extract applied) cell group)_/(570 nm-650 nm) _(control (no extract applied) cell group)_] × 100. IC_50_ was calculated based on logarithmic cell viability percentages.

#### Flow cytometric apoptosis analysis

Apoptotic changes in the plasma membrane were evaluated after 24-h treatment with 5 µg/mL of each extract using the Annexin V-FITC/PI Apoptosis Detection Kit (E-CK-A211, Elabscience), following the manufacturer’s protocol. Briefly, treated cells were harvested and transferred into 12 × 75 mm polystyrene tubes, followed by centrifugation at 1100 rpm for 5 min at room temperature (RT). The resulting cell pellets were resuspended in 1–2 mL of Annexin V Binding Buffer (AVBB) and centrifuged again under the same conditions. After aspirating the supernatant, each pellet was resuspended in 100 µL of propidium iodide (PI) working solution prepared in AVBB, and 5 µL of Annexin V-FITC was added. The samples were incubated for 15 min at RT in the dark. Following incubation, 400 µL of AVBB was added, and the samples were kept on ice until analysis. Stained cells were analyzed using a BD Accuri C6 Plus flow cytometer equipped with the appropriate laser and filter settings. Based on dual staining, cell populations were classified as: viable (Annexin V⁻/PI⁻), early apoptotic (Annexin V⁺/PI⁻), late apoptotic or secondary necrotic (Annexin V⁺/PI⁺), and necrotic (Annexin V⁻/PI⁺). Data were expressed as percentage of total cells in each quadrant [22].

#### Cell cycle analysis by propidium iodide staining

The cell cycle assay was performed as previously described [23]. Briefly, 12 × 75 mm centrifuge tubes were pre-filled with 4.5 mL of 70% ethanol and kept on ice. Approximately 10^6^ to 10^7^ cells were harvested and suspended in 5 mL of phosphate-buffered saline (PBS), then centrifuged at ~ 200 × g (1000 rpm) for 6 min. The resulting pellet was gently resuspended in 0.5 mL of PBS using a Pasteur pipette to ensure a single-cell suspension, which is essential to prevent irreversible cell aggregation during fixation. The suspension was then transferred to the pre-chilled ethanol tubes and incubated at + 4 °C for a minimum of 2 h for fixation.

Following fixation, the cells were centrifuged for 5 min at 200 × g and the ethanol was carefully decanted. The pellet was resuspended in 5 mL of PBS, incubated for 60 s, and centrifuged again under the same conditions. Finally, the cells were resuspended in 1 mL of PI staining solution containing RNase A, and incubated either for 15 min at 37 °C or for 30 min at room temperature in the dark. Cell cycle distribution was analyzed using a BD Accuri C6 Plus flow cytometer equipped with appropriate laser and filter settings.

#### Quantification of p21 and cleaved caspase-3 protein levels

Total cellular proteins were extracted from cultured cells treated with 5 µg/mL of the extracts using RIPA lysis buffer (Thermo Fisher Scientific, USA). The total protein concentration was determined spectrophotometrically by the BCA assay (Pierce, Thermo Fisher Scientific, USA). Equal amounts of protein, approximately 40–50 µg per sample, were then analyzed using sandwich ELISA kits according to the manufacturers’ instructions. Human Total p21/CIP1/CDKN1A DuoSet IC ELISA (R&D Systems, USA) and Human/Mouse Cleaved Caspase-3 (Asp175) DuoSet IC ELISA (R&D Systems, USA) kits were employed for the quantification of p21 and cleaved caspase-3, respectively. Protein concentrations were calculated from standard curves generated as per the kit guidelines, and the results were expressed as picograms per microliter (pg/µL).

#### Antimicrobial activity assay

The antibacterial and antifungal activities of *U. dioica* extracts were evaluated using the disc diffusion method. The tested microorganisms included four bacterial strains—*Enterococcus faecalis* (ATCC 29212), *Escherichia coli* (ATCC 25922), *Staphylococcus aureus* (ATCC 29213), and *Pseudomonas aeruginosa* (ATCC 27853)—and two fungal strains—*Candida albicans* (SC5314/ATCC MYA-2876) and *Candida glabrata* (ATCC 2001).

For the assay, 100 mg of each extract (ME and HE) was dissolved in 100% DMSO to obtain a stock solution at a concentration of 10 μg/μL. From this solution, 100 μL was loaded onto sterile paper discs (6 mm diameter), which were then placed onto agar plates previously inoculated with test microorganisms. Bacterial suspensions (~ 1 × 10^8^ CFU/mL) were prepared in LB broth, while fungal suspensions (~ 1 × 10^7^ CFU/mL) were prepared in YPD broth. The inoculated media were evenly spread onto Petri dishes (90 mm in diameter) under aseptic conditions. The plates were incubated at 37 °C for 24 h. Ampicillin (100 μg/disc) was used as the positive control for bacteria, and caspofungin (100 μg/disc) for fungi. Discs loaded with DMSO alone served as negative controls. After incubation, antimicrobial activity was determined by measuring the diameter of the inhibition zones (in millimeters) surrounding each disc.

### Statistical analysis

All experiments were performed in triplicate, and the results are expressed as mean ± standard deviation (SD). Statistical analyses were conducted to assess the significance of differences between groups. For the evaluation of TPC, TFC, and antioxidant activity (DPPH and CUPRAC assays), Student’s t-test was applied to compare the two extraction groups (ME and HE). For in vitro cytotoxicity data obtained from multiple treatment groups across different cell lines, two-way analysis of variance (ANOVA) followed by Tukey’s post hoc test was used to determine significant differences between treatments. Prior to ANOVA, data normality was assessed using the Shapiro–Wilk test, and homogeneity of variances was confirmed via Levene’s test. Statistical significance was set at *p* < *0.05*.

## Results

### Effect of solvent polarity on TPC, TFC, and antioxidant activity in U. dioica

The TPC, TFC, and antioxidant activities of *U. dioica* extracts were determined to evaluate the influence of solvent polarity on extraction efficiency. TPC and TFC were measured as indicators of phytochemical yield. The ME exhibited significantly higher values for both TPC and TFC compared to the HE (*p* < *0.001* for both).

**Fig. 1:**
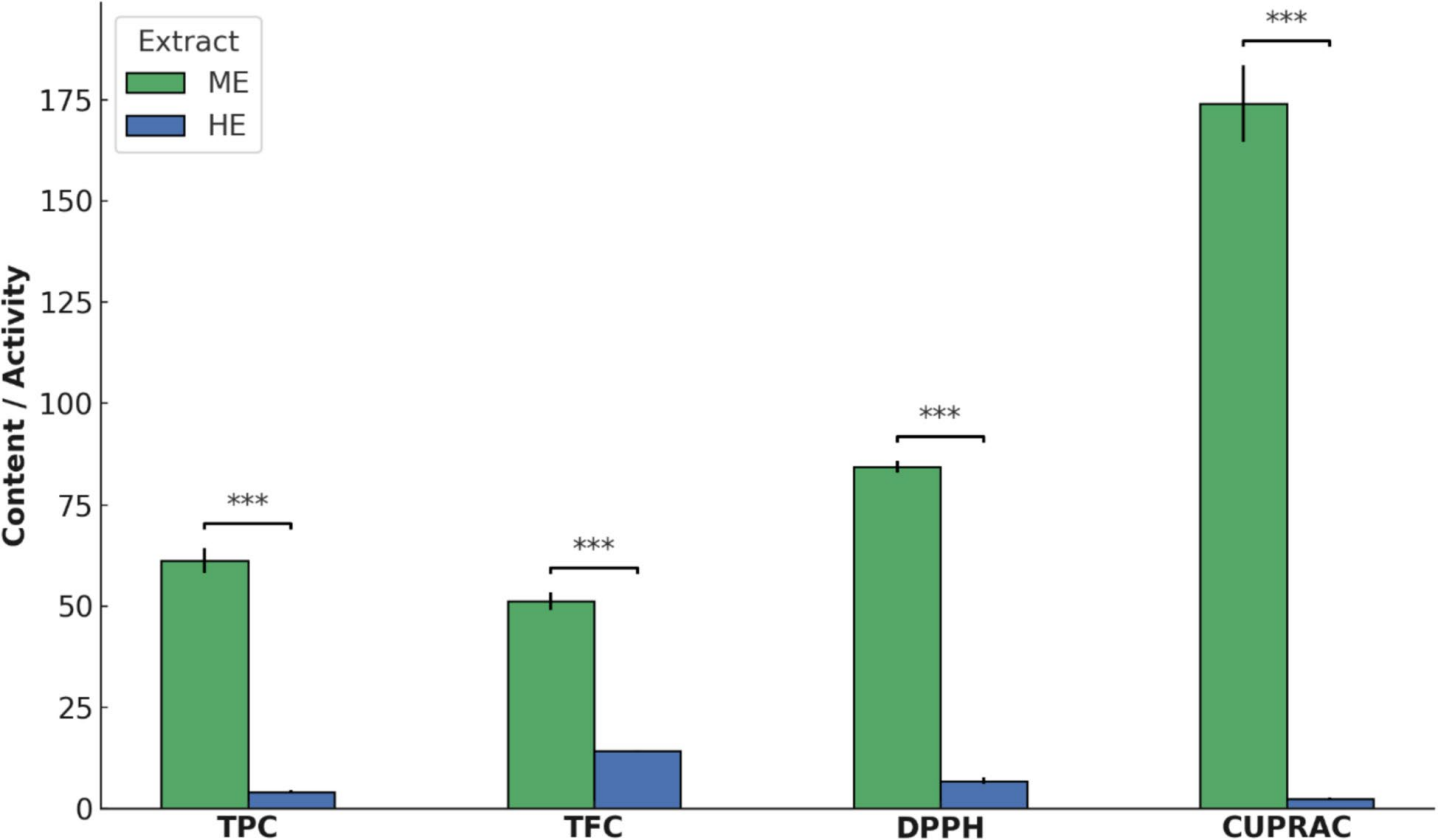
Total phenolic and flavonoid contents and antioxidant activities of acidified methanol (ME) and hexane (HE) extracts (1 mg/mL) of *U. dioica*. TPC, mg gallic acid equivalent (GAE)/g) and TFC, mg catechin equivalent (CE)/g). Antioxidant activity was assessed by DPPH radical scavenging capacity and CUPRAC reducing power, both expressed as mg Trolox equivalent (TE)/g. Bars represent the mean ± standard deviation of three replicates. Statistical significance between ME and HE for each parameter was determined using independent t-tests. Asterisks indicate statistically significant differences (***p < *0.001*)

The TPC of ME was found to be 61.25 ± 3.07 mg GAE/g, while HE showed a considerably lower value of 4.14 ± 0.47 mg GAE/g. Similarly, the TFC was 51.20 ± 2.01 mg CE/g in ME and 14.24 ± 0.21 mg CE/g in HE (Table 1, Fig. 1).

**Table 1:** TPC, TFC, and antioxidant activities of *U. dioica* extracts at 1 mg/mL concentration

	**TPC (mg GAE/g)**	**TFC (mg CE/g)**	**DPPH (mg TE/g)**	**CUPRAC (mg TE/g)**
ME	61.25 ± 3.07^a^	51.20 ± 2.01^a^	84.36 ± 1.50^a^	174.04 ± 9.54^a^
HE	4.14 ± 0.47^b^	14.24 ± 0.21^b^	6.86 ± 0.80^b^	2.36 ± 0.35^b^
*p* Value	< *0.001*	< *0.001*	< *0.001*	< *0.001*

*TPC* Total phenolic content, *TFC* Total flavonoid content, *DPPH* 2,2-Diphenyl-1-picrylhydrazyl, *CUPRAC* Cupric Ion Reducing Antioxidant Capacity, *ME* Acidified methanolic extract, *HE* Hexane extract, *mg GAE/g* milligrams of gallic acid equivalent per gram, *mg CE/g* milligrams of catechin equivalent per gram, *mg TE/g* milligrams of Trolox equivalent per gram

Different superscript letters (a, b) in the same column indicate statistically significant differences (*p* < *0.05*)

Antioxidant activity was assessed using two complementary in vitro assays: DPPH radical scavenging activity and CUPRAC (cupric ion reducing antioxidant capacity). The ME showed significantly higher antioxidant activity in both assays compared to HE (*p* < *0.001* for both). Specifically, DPPH values were 84.36 ± 1.50 mg TE/g for ME and 6.86 ± 0.80 mg TE/g for HE. In the CUPRAC assay, ME recorded 174.04 ± 9.54 mg TE/g, while HE showed a value of 2.36 ± 0.35 mg TE/g (Table 1, Fig. 1). Complete dose–response data for each extract and cell line are presented in Supplementary Table S1.

### In vitro* cytotoxic activity*

**Fig. 2:**
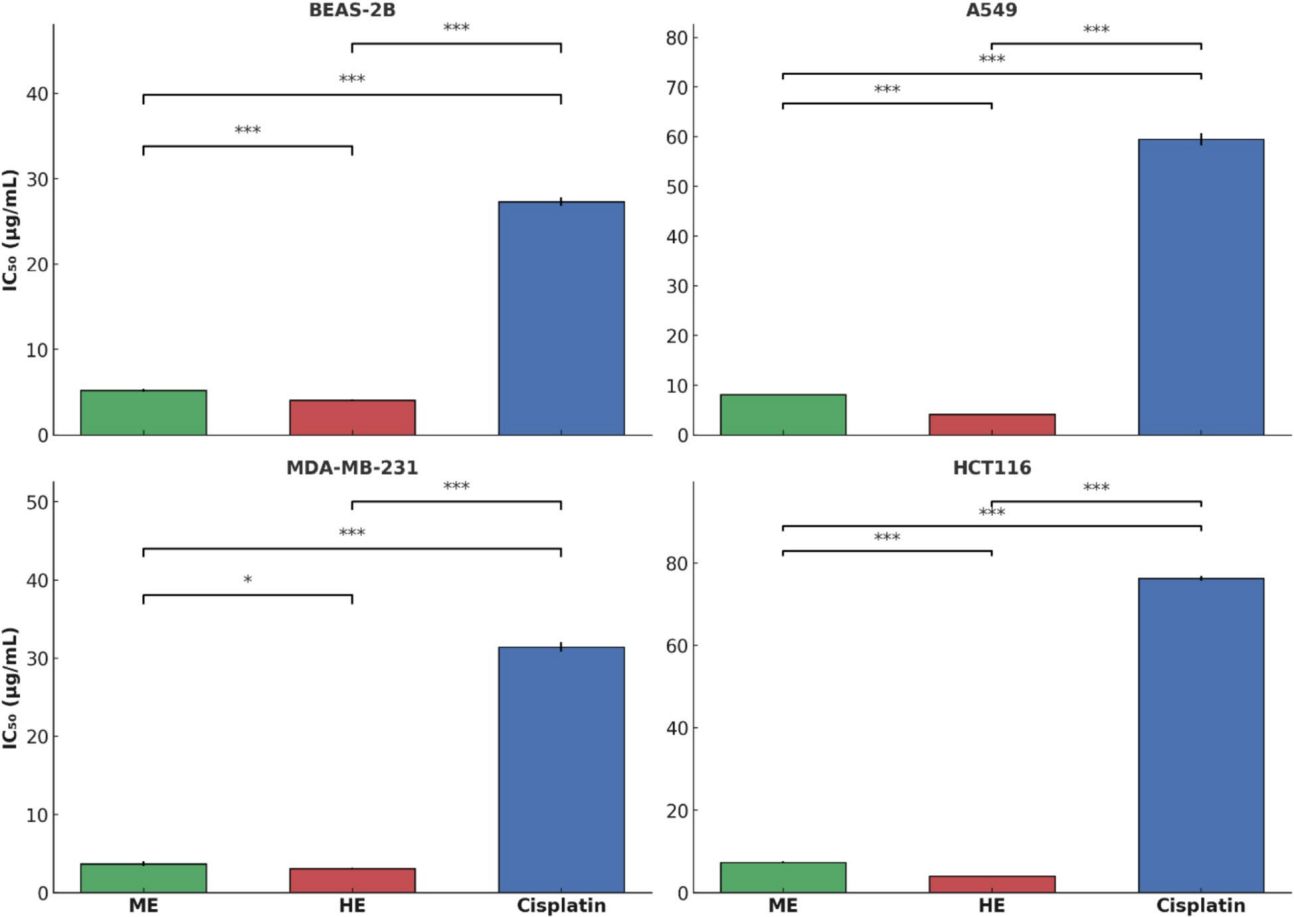
The cytotoxic effects of acidified methanol extract (ME), hexane extract (HE), and cisplatin on four different cell lines. The bar plots represent the mean IC₅₀ values (µg/mL) ± standard deviation (SD) from three independent experiments (*n* = 3). Statistical comparisons between treatment groups within each cell line were performed using independent t-tests. Asterisks indicate statistically significant differences (*p < *0.05*, **p < *0.01*, ***p < *0.001*)

The cytotoxic effects of the *U. dioica* ME, HE, and the standard chemotherapeutic agent cisplatin were evaluated in three human cancer cell lines (A549, MDA-MB-231, HCT116) and one healthy bronchial epithelial cell line (BEAS-2B) using IC_50_ values (µg/mL). The results are summarized in Table 3 and Fig. 2.

A two-way ANOVA was performed to evaluate the effects of treatment type (ME, HE, cisplatin), cell line (BEAS-2B, A549, MDA-MB-231, HCT116), and their interaction on IC_50_ values. The analysis revealed statistically significant main effects for both treatment (F(2, 24) = 34,733.62, *p* < *0.001*) and cell line (F(3, 24) = 2901.01, *p* < *0.001*), as well as a significant interaction effect between treatment and cell line (F(6, 24) = 2292.64, *p* < *0.001*), indicating that the cytotoxic effect of each treatment varied depending on the cell line (Table 2).

**Table 2:** Two-Way ANOVA results for the effects of in vitro cytotoxic activity of *U. dioica* extracts

	**Sum_sq**	**df**	**F**	**Sig**
Treatment	15,300.547	2.0	34,733.623	< *0.001*
Cell line	1916.891	3.0	2901.010	< *0.001*
Treatment* Cell line	3029.798	6.0	2292.638	< *0.001*
Residual	5.286	24.0		

Post-hoc multiple comparisons using the Tukey HSD test further revealed statistically significant differences between various treatment-cell line combinations (*p* < *0.05*). IC_50_ values for both ME and HE were significantly lower than those of cisplatin across all tested cell lines, indicating stronger cytotoxic activity of the plant extracts under the experimental conditions. For instance, HE showed significantly lower IC_50_ than cisplatin in A549 cells (mean difference = − 55.39, *p* < *0.001*), while ME demonstrated greater cytotoxicity compared to cisplatin in both HCT116 and MDA-MB-231 cell lines.

Moreover, significant differences were also observed between cisplatin-treated groups depending on the cell line, suggesting variable sensitivity of different cell types to the standard chemotherapeutic agent. Both ME and HE exhibited comparable or even stronger cytotoxicity in BEAS-2B non-cancerous cells (IC50 = 5.22 ± 0.17 µg/mL and 4.08 ± 0.10 µg/mL, respectively) than in some cancer cell lines, suggesting limited selectivity (Table 3).

**Table 3:** IC_50_ values (µg/mL) of *U. dioica* extracts and cisplatin on cancer and healthy cell lines

	BEAS-2B*	A549*	MDA-MB-231*	HCT116*
ME	5.22 ± 0.17^b^	8.09 ± 0.06^b^	3.68 ± 0.32^b^	7.35 ± 0.25^b^
HE	4.08 ± 0.10^c^	4.12 ± 0.06^c^	3.10 ± 0.11^b^	4.01 ± 0.12^c^
Cisplatin **	27.30 ± 0.51^a^	59.51 ± 1.20^a^	31.44 ± 0.59^a^	76.33 ± 0.59^a^
*p* Value	< *0.001*	< *0.001*	< *0.001*	< *0.001*

^*^ Cell lines, ^**^ Reference drug, ME: Acidified methanolic extract, HE: Hexane extract

IC_50_ values presented as mean ± SD of three independent experiments. Different lowercase letters (a-c) within a column indicate statistically significant differences (*p* < *0.05*)

### Flow cytometric evaluation of apoptosis

**Fig. 3:**
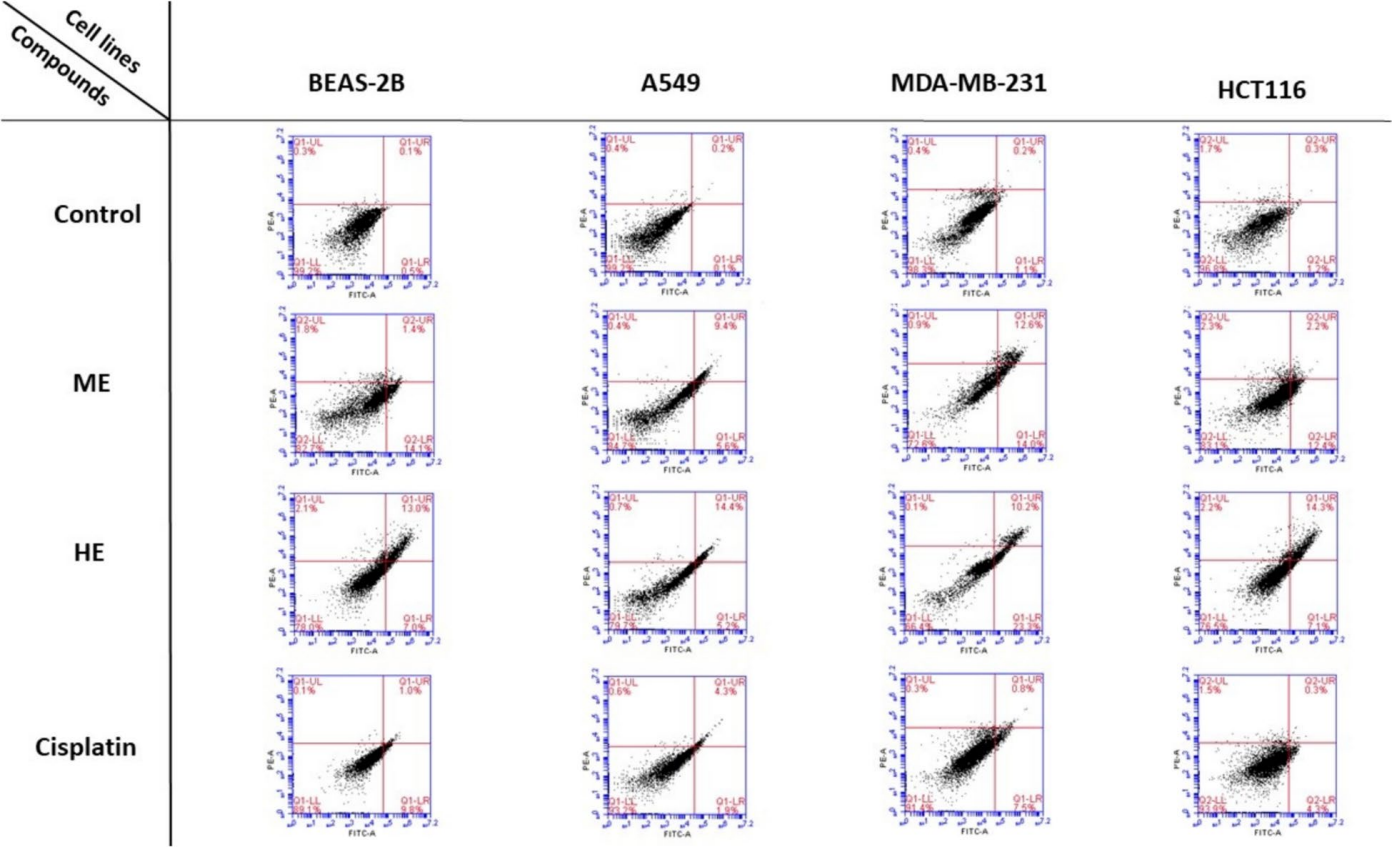
Flow cytometry dot plots showing apoptosis profiles in BEAS-2B, A549, MDA-MB-231, and HCT116 cells after 24-h treatment with *U. dioica* extracts (ME, HE) and cisplatin, using Annexin V-FITC/PI staining (ME: Acidified methanolic extract, HE: Hexane extract)

The apoptotic effects of *U. dioica* ME, HE, and cisplatin were assessed in BEAS-2B, A549, MDA-MB-231, and HCT116 cell lines using Annexin V-FITC/PI dual staining followed by flow cytometric analysis. Cells were treated with 5 µg/mL of each extract for 24 h, and the percentage of total apoptosis (early + late apoptotic cells) was quantified (Table 4, Fig. 3).

**Table 4:** Percentage of total apoptotic cells (early + late apoptosis) in four cell lines (BEAS-2B, A549, MDA-MB-231, HCT116) following 24-h treatment with *U. dioica* ME, HE, and cisplatin

	BEAS-2B*	A549*	MDA-MB-231*	HCT116*
Control	0.67 ± 0.21^d^	0.43 ± 0.15^d^	1.10 ± 0.20^d^	1.07 ± 0.21^d^
ME	16.93 ± 0.35^b^	15.30 ± 0.30^b^	25.47 ± 1.06^b^	13.87 ± 0.70^b^
HE	20.27 ± 0.31^a^	19.77 ± 0.29^a^	32.77 ± 0.67^a^	20.87 ± 0.50^a^
Cisplatin **	10.63 ± 0.67^c^	6.10 ± 0.26^c^	8.10 ± 0.26^c^	4.40 ± 0.26^c^
*p* Value	< *0.001*	< *0.001*	< *0.001*	< *0.001*

^*^: Cell lines, ^**^: Reference drug, ME: Acidified methanolic extract, HE: Hexane extract

Data are presented as mean ± standard deviation (SD) from three independent experiments. All treatment groups showed significantly higher apoptosis rates compared to the untreated control group (*p* < *0.001*)

Different superscript letters (a-d) in the same column indicate statistically significant differences (*p* < *0.05*)

As summarized in Table 4, both ME and HE significantly increased apoptosis in all tested cell lines compared to the untreated controls (*p* < *0.001*). HE treatment resulted in the highest apoptotic response across all cancer cell lines. In MDA-MB-231 cells, HE induced the most pronounced effect (mean apoptosis: 32.77 ± 0.67%), followed by ME (25.47 ± 1.06%) and cisplatin (8.10 ± 0.26%). Similarly, in A549 cells, HE (19.77 ± 0.29%) and ME (15.30 ± 0.30%) showed significantly stronger pro-apoptotic effects than cisplatin (6.10 ± 0.26%).

In HCT116 cells, both plant extracts triggered higher apoptotic rates compared to cisplatin, with HE inducing 20.87 ± 0.50% and ME 13.87 ± 0.70%, whereas cisplatin resulted in only 4.40 ± 0.26% apoptosis. Notably, even in non-cancerous BEAS-2B cells, ME (16.93 ± 0.35%) and HE (20.27 ± 0.31%) led to greater apoptotic induction than cisplatin (10.63 ± 0.67%), although overall levels remained lower than those observed in malignant cells.

Statistical analysis confirmed that these differences were highly significant (*p* < *0.001*), highlighting the potent pro-apoptotic capacity of both *U. dioica* extracts, particularly HE, across multiple cell lines.

### Effects on cell cycle distribution

**Fig. 4:**
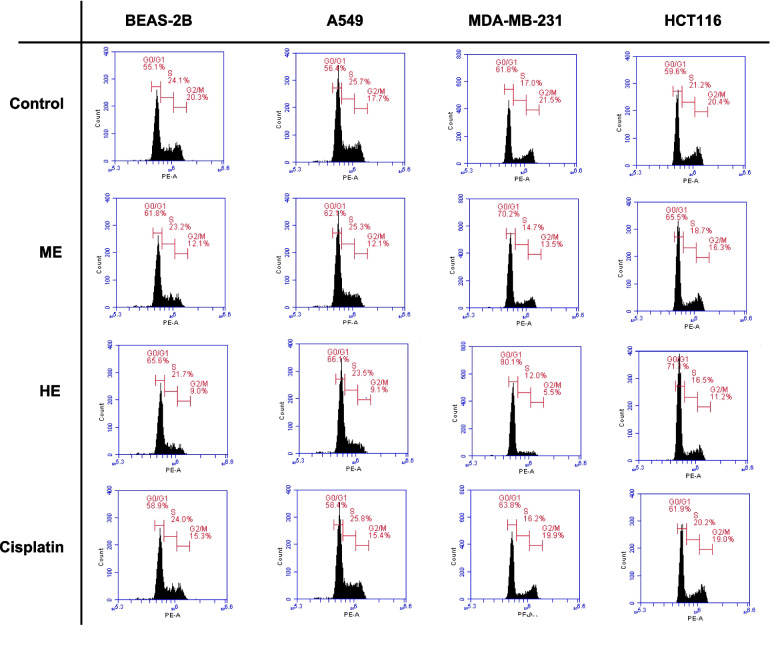
Representative cell cycle histograms of BEAS-2B, A549, MDA-MB-231, and HCT116 cell lines after 24 h treatment with acidified methanolic extract (ME), hexane extract (HE), or cisplatin (positive control). Cell populations in G0/G1, S, and G2/M phases were quantified by flow cytometry. Percentages for each phase are shown. One representative histogram from three independent experiments is presented (*n* = 3)

The influence of *U. dioica* extracts and cisplatin on cell cycle progression was assessed in BEAS-2B, A549, MDA-MB-231, and HCT116 cell lines using flow cytometric analysis after PI staining. The percentages of cells in G0/G1, S, and G2/M phases were quantified following 24-h treatment with ME, HE, or cisplatin, and results are summarized in Table 5 and visualized in Fig. 4.

**Table 5:** Distribution (%) of cells in G0/G1, S, and G2/M phases following 24-h treatment with *U. dioica* ME, HE, and cisplatin in BEAS-2B, A549, MDA-MB-231, and HCT116 cell lines

	BEAS-2B*	A549*	MDA-MB-231*	HCT116*
		**G0/G1**		
Control	55.57 ± 0.45^d^	56.13 ± 0.25^d^	61.40 ± 0.46^d^	59.70 ± 0.36^d^
ME	61.33 ± 0.45^b^	62.07 ± 0.06^b^	70.60 ± 0.40^b^	65.73 ± 0.32^b^
HE	65.93 ± 0.29^a^	66.17 ± 0.31^a^	80.30 ± 0.53^a^	71.37 ± 0.55^a^
Cisplatin**	58.93 ± 0.15^c^	58.90 ± 1.04^c^	63.80 ± 0.30^c^	62.53 ± 0.93^c^
*p* Value	< *0.001*	< *0.001*	< *0.001*	< *0.001*
		**S**		
Control	23.57 ± 0.55^a^	25.70 ± 0.60^a^	17.13 ± 0.91^a^	21.13 ± 0.21^a^
ME	23.27 ± 0.31^a^	25.13 ± 0.15^ab^	14.07 ± 0.60^b^	18.37 ± 0.42^b^
HE	21.27 ± 0.45^b^	23.53 ± 0.55^b^	11.90 ± 0.46^c^	16.20 ± 0.36^c^
Cisplatin **	23.77 ± 0.49^a^	25.23 ± 1.25^ab^	16.80 ± 0.53^a^	18.70 ± 1.45^b^
*p* Value	= *0.005*	= *0.0346*	< *0.001*	= 0.005
		**G2/M**		
Control	19.67 ± 0.71^a^	17.77 ± 0.31^a^	21.73 ± 0.32^a^	20.30 ± 0.36^a^
ME	12.23 ± 0.32^c^	12.43 ± 0.31^c^	13.23 ± 0.31^c^	16.27 ± 0.15^c^
HE	8.93 ± 0.21^d^	9.13 ± 0.15^d^	5.60 ± 0.36^d^	11.23 ± 0.25^d^
Cisplatin **	15.63 ± 0.35^b^	14.60 ± 1.22^b^	19.33 ± 0.55^b^	18.80 ± 0.26^b^
*p* Value	< *0.001*	< *0.001*	< *0.001*	< *0.001*

^*^: Cell lines, ^**^: Reference drug, ME: Acidified methanolic extract, HE: Hexane extract

Data are presented as mean ± standard deviation (SD) from three independent experiments. Different superscript letters (a-d) in the same column indicate statistically significant differences (*p* < *0.05*)

Treatment with HE resulted in a significant accumulation of cells in the G0/G1 phase across all cell lines compared to untreated controls (*p* < *0.001*), indicating a potential cell cycle arrest at this checkpoint. For example, HE-treated MDA-MB-231 cells exhibited a G0/G1 proportion of 80.30 ± 0.53%, significantly higher than the control value of 61.40 ± 0.46%. Similarly, HE increased G0/G1 populations in BEAS-2B (65.93 ± 0.29%) and A549 (66.17 ± 0.31%) cells.

Correspondingly, a significant decrease in the G2/M phase population was observed in all extract-treated groups. In MDA-MB-231 cells, HE reduced the G2/M population to 5.60 ± 0.36%, compared to 21.73 ± 0.32% in the control group (*p* < *0.001*). ME treatment also induced a moderate G0/G1 arrest and reduced G2/M percentages in all cell lines. In contrast, cisplatin exhibited a more balanced distribution across phases but was generally less effective than HE in inducing G0/G1 arrest.

The S phase population showed a slight but statistically significant decrease in extract-treated cells, particularly with HE, supporting a general slowing of cell cycle progression. These findings suggest that *U. dioica* extracts, especially HE, exert anti-proliferative effects through G0/G1 phase arrest, which may underlie their cytotoxic activity.

### Effects on p21 and cleaved caspase-3 Levels

To explore potential molecular mechanisms associated with the cytotoxic and apoptotic effects of *U. dioica* extracts, intracellular p21 and cleaved caspase-3 levels were measured in BEAS-2B, A549, MDA-MB-231, and HCT116 cells after 24-h exposure to the ME, HE, and cisplatin. Both ME and HE treatments were associated with an increase in p21 protein concentrations compared with control and cisplatin-treated cells. The elevation was approximately 4 – sixfold across cell lines, with relatively higher values observed in HE-treated groups (mean range: 3.55—3.93 pg/µL vs. 0.63—0.94 pg/µL in controls). Cleaved caspase-3 levels were also significantly elevated following treatment, suggesting activation of apoptotic processes. The increase was more pronounced in HE-treated MDA-MB-231 cells (~ 10 pg/µL vs. ~ 1.2 pg/µL in controls), corresponding to an approximately eightfold change (Table 6). Both extracts showed higher cleaved caspase-3 values than cisplatin under the tested conditions.

**Table 6:** Intracellular p21 and cleaved caspase-3 levels (pg/µL) in BEAS-2B, A549, MDA-MB-231, and HCT116 cells following treatment with ME, HE, and cisplatin (5 µg/mL for 24 h)

	BEAS-2B*	A549*	MDA-MB-231*	HCT116*
	**p21 Level (pg/µL)**			
Control	0,68 ± 0.11^c^	0,94 ± 0,07^d^	0,71 ± 0,05^d^	0,63 ± 0,04^d^
ME	3,33 ± 0.13^a^	2,52 ± 0,09^b^	3,76 ± 0,08^b^	2,73 ± 0,08^b^
HE	3,55 ± 0.09^a^	3,73 ± 0,09^a^	3,93 ± 0,03^a^	3,63 ± 0,13^a^
Cisplatin**	1,47 ± 0.10^b^	1,27 ± 0,08^c^	1,43 ± 0,06^c^	1,16 ± 0,05^c^
*p* Value	< *0.001*	< *0.001*	< *0.001*	< *0.001*
	**Cleaved Caspase 3 Level (pg/µL)**			
Control	1,25 ± 0,06^d^	1,06 ± 0,09^d^	1,24 ± 0,08^d^	1,15 ± 0,07^d^
ME	7,45 ± 0,13^b^	4,69 ± 0,10^b^	9,02 ± 0,10^b^	4,95 ± 0,17^b^
HE	7,94 ± 0,02^a^	7,73 ± 0,07^a^	9,96 ± 0,14^a^	7,46 ± 0,29^a^
Cisplatin **	2,64 ± 0,08^c^	2,31 ± 0,10^c^	2,44 ± 0,09^c^	2,21 ± 0,07^c^
*p* Value	< *0.001*	< *0.001*	< *0.001*	< *0.001*

^*^: Cell lines, ^**^: Reference drug, ME: Acidified methanolic extract, HE: Hexane extract

Data are presented as mean ± standard deviation (SD) from three independent experiments. Different superscript letters (a-d) in the same column indicate statistically significant differences (*p* < *0.05*)

### Antimicrobial activity

**Fig. 5:**
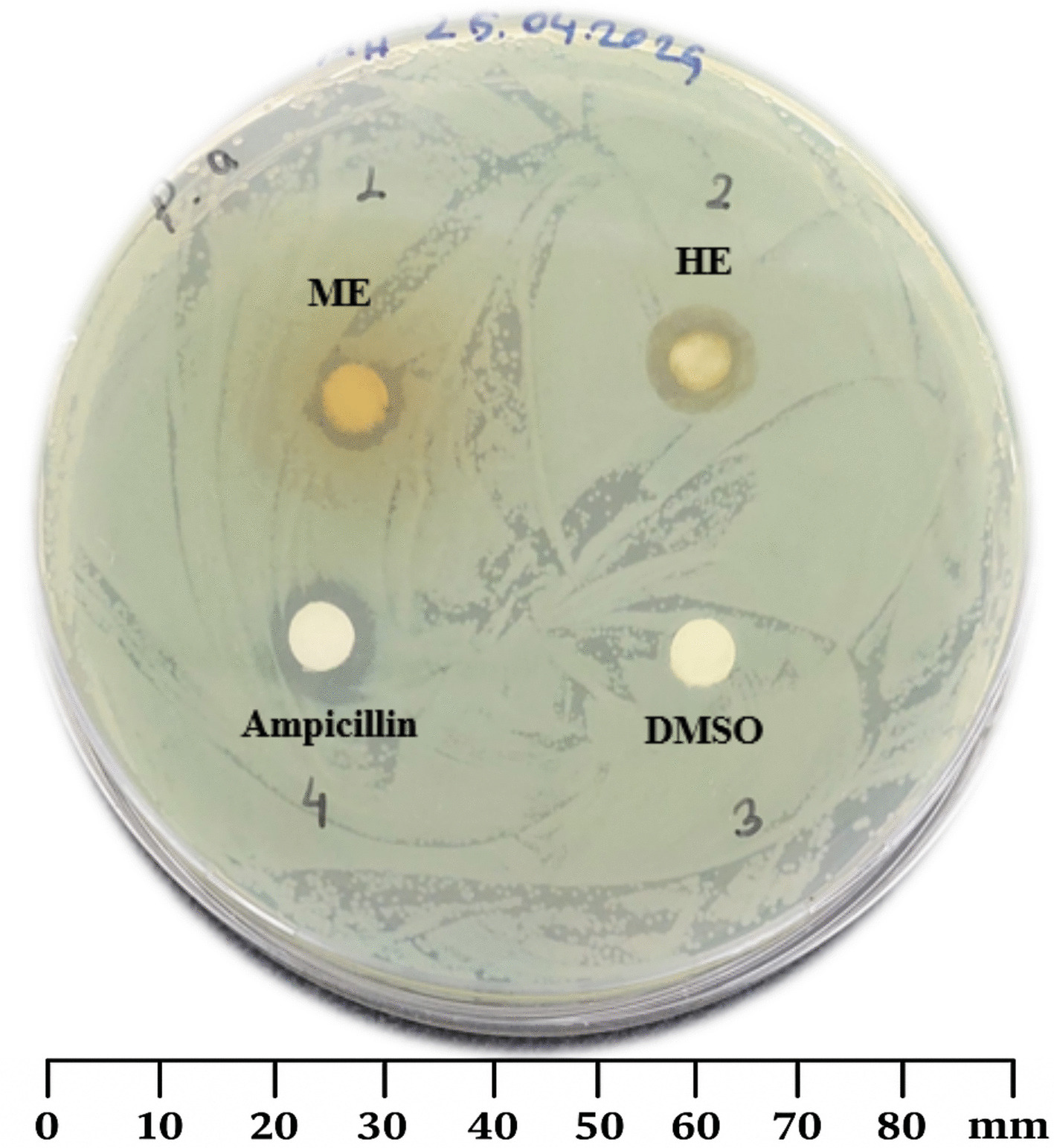
Antibacterial activity of *U. dioica* ME and HE extracts against *P. aeruginosa* using the disc diffusion method. (1: Acidified methanolic extract (ME), 2: Hexane extract, 3: Ampicillin as positive control, and 4: DMSO as negative control)

The antimicrobial effects of *U. dioica* ME, HE, and reference antibiotics were assessed against six microbial strains, including fungi (*C. albicans, C. glabrata*), Gram-negative bacteria (*E. coli, P. aeruginosa*), and Gram-positive bacteria (*S. aureus, E. faecalis*), using the disc diffusion method. The results, presented as inhibition zone diameters (mm), are summarized in Fig. 5 and Table 7.

**Table 7:** Inhibition zone diameters (mm) representing the antimicrobial activity of *U. dioica* ME, HE, and standard antibiotics (caspofungin and ampicillin) against selected microbial strains

Samples	Inhibition zone (mm)				
	**ME**	**HE**	**Caspofungin**	**Ampicillin**	***p* Value**
*C. albicans*	NI	9.90 ± 0.10^b^	26.63 ± 0.42^a^	NI	< *0.001*
*C. glabrata*	7.17 ± 0.35^b^	8.30 ± 0.20^b^	29.17 ± 0.86^a^	NI	< *0.001*
*E. coli*	NI	7.50 ± 0.46^b^	NI	20.27 ± 0.15^a^	< *0.001*
*P. aeruginosa*	7.93 ± 0.74^b^	9.97 ± 0.15^a^	NI	9.60 ± 0.46^a^	< *0.001*
*S. aureus*	9.27 ± 0.70^b^	10.00 ± 0.10^b^	NI	24.47 ± 0.65^a^	< *0.001*
*E. faecalis*	NI	6.77 ± 0.21^b^	NI	22.23 ± 0.23^a^	< *0.001*

*ME* Acidified methanolic extract, *HE* Hexane extract, *NI* Not Inhibition. Results are presented as mean ± standard deviation (SD) from three independent experiments. Different superscript letters (a, b) in the same column indicate statistically significant differences (*p* < *0.05*)

The HE extract showed measurable antimicrobial activity against all tested strains, with inhibition zones ranging from 6.77 ± 0.21 mm (*E. faecalis*) to 10.00 ± 0.10 mm (*S. aureus*). While the inhibition was modest in magnitude, the activity spanned both fungal and bacterial species, indicating a relatively broad target range. In contrast, the ME extract demonstrated limited and selective activity, inhibiting only *C. glabrata* (7.17 ± 0.35 mm), *P. aeruginosa* (7.93 ± 0.74 mm), and *S. aureus* (9.27 ± 0.70 mm), with no detectable zones against *C. albicans*, *E. coli*, or *E. faecalis*.

Caspofungin, used as the antifungal control, showed strong activity against *C. albicans* (26.63 ± 0.42 mm) and *C. glabrata* (29.17 ± 0.86 mm). Ampicillin, the antibacterial control, displayed expected efficacy, with significant inhibition zones against *E. coli* (20.27 ± 0.15 mm), *P. aeruginosa* (9.60 ± 0.46 mm), *S. aureus* (24.47 ± 0.65 mm), and *E. faecalis* (22.23 ± 0.23 mm). All differences between groups were statistically significant (*p* < *0.001*).

These findings suggest that while the ME extract exhibits selective and modest antimicrobial activity, the HE extract demonstrates broader, though still moderate, antimicrobial potential across different classes of pathogens. Nonetheless, the inhibition zones remain substantially smaller than those produced by standard antibiotics, indicating that *U. dioica* extracts may be more suitable as complementary rather than standalone antimicrobial agents.

## Discussion

The present study aimed to evaluate the total phenolic and flavonoid contents and biological activities of *U. dioica* extracts obtained using two solvents with differing polarities—methanol and hexane. The results revealed that solvent polarity had a profound effect on both the chemical profile and bioactivity of the extracts.

In the total phenolic and flavonoid contents analyses, ME exhibited significantly higher levels of TPC and TFC compared to HE. This outcome is consistent with previous findings [9] suggesting that polar solvents such as methanol are more efficient in extracting hydrophilic phenolic compounds and flavonoids, whereas non-polar solvents like hexane tend to extract lipophilic constituents with lower antioxidant potential. In antioxidant capacity assays, the ME consistently demonstrated superior activity over the HE, as evidenced by both DPPH and CUPRAC methods. These results are broadly consistent with previously reported findings, while also reflecting methodological and solvent-related variations. In line with this, methanol extracts of *U. dioica* have been reported to possess stronger antioxidant properties than non-polar extracts, as demonstrated by Çolak et al. [24] and Gülçin et al. [25].

Importantly, LC–MS/MS analysis of the same plant material performed in our previous work identified a number of phenolic compounds in the methanolic extract, including acetohydroxamic acid (52.54 ± 1.88 mg/100 g), gallic acid (2.80 ± 0.03 mg/100 g), caffeic acid (13.48 ± 0.18 mg/100 g), ellagic acid (6.21 ± 0.60 mg/100 g), and quercetin (2.31 ± 0.08 mg/100 g), with several other compounds detected below the quantification limit [4]. Among these, acetohydroxamic acid was the most abundant, while caffeic acid, ellagic acid, and quercetin are well-documented antioxidants known to neutralize free radicals, inhibit ROS-mediated cellular damage, and modulate redox-sensitive pathways [26–30]. These phenolics likely contributed to the high radical scavenging capacity observed in ME. Similarly, Jeszka-Skowron et al. [31] confirmed the presence of syringic acid, protocatechuic acid, p-coumaric acid, ferulic acid, kaempferol, quercetin, and rutin in different parts of *U. dioica*, further reinforcing the role of phenolics in the antioxidant potential of methanol extracts.

In contrast, the HE, despite its lower phenolic content, exhibited stronger cytotoxic effects on cancer cell lines (A549, MDA-MB-231, and HCT116), suggesting the presence of non-polar cytotoxic constituents selectively extracted by hexane. Previous GC–MS analyses support this interpretation. Dar et al. [8] identified major compounds in *U. dioica* hexane extracts, including neophytadiene (19.96%), 2,6,10,15-tetramethylheptadecane (12.82%), heptadecyl ester (9.45%), butyl etradecyl ester (9.53%), hexyl octyl ester (6.31%), and phthalic acid derivatives (9.89%), along with minor fractions such as long-chain hydrocarbons, benzoic acid derivatives, and phenolic antioxidants (e.g., 2,4-di-tert-butylphenol). Similarly, Grauso et al. [32] reported high levels of fatty acids (palmitic, oleic, stearic, arachidic, behenic, lignoceric, cerotic) and triterpenoids (α-amyrin, 10.9%; β-amyrin, 9.7%) in hexane extracts of *U. dioica*. These lipophilic constituents are associated with cytotoxic, antimicrobial, and anti-inflammatory properties.

Overall, these observations align with the broader literature comparing polar and non-polar extracts of *U. dioica*. Omer and Mohammed [9] reported that the ethanol extract exhibited superior results (TPC: 359.71 mg TAE/g, TFC: 215.17 mg QE/g, DPPH IC_50_: 15.92 µg/mL) compared to the hexane extract (TPC: 136.63 mg TAE/g, TFC: 135.89 mg QE/g, DPPH IC_50_: 125.62 µg/mL), further highlighting the efficacy of polar solvents in extracting antioxidant compounds. Moreover, previous studies have shown that the TPC and TFC values of *U. dioica* extracts vary widely depending on the extraction technique and solvent used, ranging from 11.21 to 359.71 mg/g for phenolic content and from 1.84 to 135.89 mg/g for flavonoid content, when expressed in terms of various phenolic acid and flavonoid standards [9, 33–37].

Interestingly, the TFC value measured in the hexane extract was slightly higher than the corresponding TPC value, which may appear contradictory given that flavonoids are a subclass of phenolic compounds. However, this result is not unprecedented and can be attributed to the limitations of the AlCl_3_ colorimetric assay used for flavonoid quantification [38]. In extracts obtained with non-polar solvents such as hexane extract, some lipophilic components (e.g. sterols, terpenoids) may react with AlCl_3_ and behave like flavonoids, thus inflating the TFC value.

In terms of cytotoxic effects, both the ME and HE of *U. dioica* demonstrated substantial in vitro activity across cancer cell lines A549, MDA-MB-231, and HCT116, with HE exhibiting consistently stronger cytotoxicity. The IC_50_ values for HE ranged from 3.10 to 4.12 µg/mL, which were significantly lower than those observed for ME, indicating more potent antiproliferative effects. This finding may reflect the presence of non-polar cytotoxic constituents selectively extracted by hexane—such as sterols and terpenoids—compounds known to exert antitumor effects and previously identified in dichloromethane or hexane extracts of *U. dioica* [39, 40]. Interestingly, although ME showed superior antioxidant capacity and higher total phenolic and flavonoid contents, this did not correlate with higher cytotoxic potency. This may be attributed to the specific phytochemical composition of the ME, as LC–MS/MS profiling revealed that key cytotoxic flavonoids such as kaempferol, luteolin, naringenin, and curcumin were below the quantification limit, potentially limiting the extract’s efficacy against cancer cells despite its antioxidant richness. Similar solvent-dependent variations in bioactivity have been reported in other medicinal plant studies as well [41, 42].

The cytotoxic effect of *U. dioica*, becomes even more apparent when compared with other studies employing similar plant species. For example, Omar and Aloqbi [43] reported that 10 µg/mL of dried or freeze-dried *U. urens* leaf extract reduced A-549 cell viability to approximately 72–74%. In our study, both ME and HE extracts of *U. dioica* achieved IC_50_ values well below 10 µg/mL in A549 cells (8.09 µg/mL for ME and 4.12 µg/mL for HE), indicating a stronger growth-inhibitory effect at comparable or lower concentrations. This superior activity may be attributed to differences in plant species (*U. dioica* vs. *U. urens*), extraction protocols, or solvent efficiency in concentrating cytotoxic constituents.

In a study conducted by Fattahi et al. [44], the aqueous extract of *U. dioica* demonstrated an antiproliferative effect on MCF-7 breast cancer cells with an IC_50_ value of 2000 µg/mL. Although a different cell line was used, this value is considerably higher compared to the IC_50_ values observed in the cell lines tested in our study, indicating a lower cytotoxic effect. This discrepancy may be attributed to differences in the extraction method and solvent type. In our study, the stronger antiproliferative effect observed with the non-polar HE extract suggests that lipophilic and cytotoxic components may have been more effectively extracted compared to aqueous extracts.

While both extracts demonstrated substantial cytotoxicity in cancer cells, their selectivity toward malignant versus normal cells remains limited. The IC_50_ values obtained for BEAS-2B cells were comparable to or even lower than those observed in cancer lines, particularly in the case of HE. To quantify this observation, Selectivity Index (SI) values were calculated as the ratio of IC_50_ in normal cells (BEAS-2B) to that in cancer cells. For HE, the SI values ranged from 0.83 (A549) to 1.10 (MDA-MB-231), suggesting minimal selectivity. Typically, SI values ≥ 2 are considered desirable for therapeutic candidates. The low SI values here indicate broad cytotoxic effects, which may be attributed to membrane-disruptive or non-specific mechanisms of lipophilic constituents [45, 46]. While this limits the systemic therapeutic potential of the extract, its strong cytotoxicity could still hold value for localized applications or as an adjuvant in combination therapies. Future studies involving compound isolation and structure–activity analysis are necessary to improve selectivity and safety profiles.

The cytotoxic effects of the extracts were further elucidated through apoptosis and cell cycle analyses. Both ME and HE significantly induced apoptosis in cancer cell lines, with HE showing a more pronounced effect. This pro-apoptotic activity was particularly notable in MDA-MB-231 cells, where HE induced 32.77% apoptosis compared to 25.47% by ME and only 8.10% by cisplatin. The cell cycle analysis revealed that HE caused a significant accumulation of cells in the G0/G1 phase, indicating a potential mechanism of action involving cell cycle arrest. This aligns with previous studies reporting G1 or G2/M phase arrest following treatment with *U. dioica* extracts [39]. Similar findings were reported by Temiz et al. [47], where *U. dioica* water extract triggered apoptosis and G0/G1 arrest in HL-60 cells. Consistent with our findings, another study reported that *U. dioica* methanolic extract exhibited antiproliferative effects on HepG2 and HCT116 cells after 48 h, with IC_50_ values of approximately 410 and 420 µg/mL, respectively, while sparing non-cancerous HDF cells. In the same study, *U. dioica* extract treatment led to increased apoptosis in HepG2 and HCT116 cells, which was accompanied by an elevated BAX/BCL-2 ratio, indicating the activation of the mitochondrial apoptotic pathway [48].

The mechanistic differences underlying the observed cytotoxic, apoptotic, and cell cycle effects between ME and HE extracts are closely linked to their distinct phytochemical compositions. LC–MS/MS profiling of the ME confirmed high levels of phenolic compounds such as acetohydroxamic acid, gallic acid, caffeic acid, ellagic acid, and quercetin [4]. These compounds are established antioxidants that regulate redox balance by scavenging ROS, inhibiting NF-κB activity, and modulating oxidative signaling pathways [49–51], which may explain ME’s antioxidant-driven, but comparatively milder cytotoxic effect.

In contrast, the more potent cytotoxic and pro-apoptotic effects of HE likely stem from its enrichment in lipophilic compounds. Literature-based GC–MS analyses of *U. dioica* hexane extracts report the presence of long-chain fatty acids (e.g., palmitic, oleic, stearic), hydrocarbons, and triterpenoids such as α- and β-amyrin [8, 32]. These lipophilic molecules are capable of: (i) disrupting cellular membranes, compromising integrity and inducing necro-apoptotic responses; (ii) inducing mitochondrial dysfunction, leading to cytochrome c release and intrinsic pathway activation [52]; (iii) triggering apoptotic signalling, as demonstrated by β-amyrin’s activation of p38/JNK and caspase-3/9 in various cancer models [46, 53, 54].

These mechanisms are in line with the G0/G1 arrest and increased apoptosis observed in HE-treated groups. While ME also induced apoptosis and cell cycle modulation, the dominant pathways may involve antioxidant-mediated redox signalling rather than direct cytotoxic stress. Therefore, ME appears to function primarily through redox modulation, whereas HE exerts its antiproliferative activity via membrane disruption, mitochondrial destabilization, and apoptosis signalling activation.

Furthermore, the observed G0/G1 cell cycle arrest, particularly in HE-treated cells, could potentially involve modulation of key regulators such as p21^Cip1/Waf1^ and p27^Kip1^, which inhibit cyclin D-CDK4/6 complexes and thereby halt progression from G1 to S phase [55]. These effects may be mediated via upstream activation of p53, a master regulator of cell cycle checkpoints in response to cellular stress. On the apoptotic front, although protein-level assays were not conducted in this study, it is plausible that the increased apoptosis observed in HE-treated cells involves an elevated BAX/BCL-2 ratio, promoting mitochondrial outer membrane permeabilization and activation of the intrinsic apoptotic pathway [56]. Supporting this, lipophilic compounds such as β-amyrin—previously identified in *U. dioica* hexane extracts—are known to activate p38/JNK signaling, enhance intracellular ROS accumulation, trigger cytochrome c release, and initiate caspase-3/9 cascades in various cancer models [54]. In support of these proposed mechanisms, the present study also evaluated the levels of key regulatory proteins associated with cell cycle control and apoptosis. Both ME and HE treatments resulted in increased expression of p21 and cleaved caspase-3 across all tested cell lines. The elevation in p21 may suggest an involvement of CDK inhibition and G0/G1 phase arrest, particularly in HE-treated groups, consistent with the flow cytometry findings. Similarly, the upregulation of cleaved caspase-3 indicates activation of the intrinsic apoptotic pathway, which may occur downstream of mitochondrial perturbation and cytochrome c release. Notably, HE induced a more pronounced increase in both markers compared to ME and cisplatin, suggesting that the stronger cytotoxicity of HE could be partially attributed to the simultaneous activation of p21-mediated cell cycle regulation and caspase-dependent apoptosis. These findings provide additional biochemical support for the hypothesis that solvent polarity not only influences the phytochemical composition of *U. dioica* extracts but also modulates distinct molecular pathways contributing to their antiproliferative effects.

In terms of antimicrobial activity, our study demonstrated that the HE of *U. dioica* exhibited moderate inhibitory effects against tested Gram-positive and Gram-negative bacteria, as well as fungi, with the highest inhibition zones observed against *C. albicans* and *P. aeruginosa*. The ME showed comparatively weaker antimicrobial activity, consistent with findings reported by Kukrić et al. [37], where ethanol extracts of *U. dioica* exhibited moderate activity against *P. aeruginosa* and *E. coli*. Similar findings were observed in a study where 80% ethanolic extracts of nettle (*U. dioica*) developed inhibition zones greater than 10 mm against *S. aureus*, *E. coli*, and *S. pyogenes*, with the highest inhibition noted against *S. pyogenes* (12.5 mm) [57]. Külcü et al. [58] investigated the antibacterial potential of ethanolic extracts of nettle and reported an inhibition zone of 7 mm against *S. aureus*, a similar zone of 7 mm against *E. faecalis*, and no observable inhibition against *E. coli*. Dar et al. [11] reported that hexane fractions of *U. dioica* exhibited inhibition zones ranging from 7 to 15 mm against *E. faecalis*, *E. coli*, *P. aeruginosa*, and *S. aureus*. Overall, our findings align with previous reports indicating that *U. dioica* extracts possess antimicrobial potential, likely attributable to their phenolic compounds, flavonoids, and terpenoids [23]. Considering that the inhibition zones remained below those produced by conventional antibiotics such as ampicillin and caspofungin, the antimicrobial effects of both ME and HE extracts should be regarded as moderate at best. These findings suggest that U. dioica extracts are unlikely to serve as stand-alone antimicrobial agents but may possess value as complementary or adjuvant components in integrated therapeutic strategies, particularly in formulations targeting multi-drug resistant organisms or in phytotherapy contexts.

Our findings are in agreement with the growing body of literature suggesting that *U. dioica* possesses both antioxidant and cytotoxic properties, but the extraction method plays a critical role in determining the spectrum and intensity of its biological effects. While polar solvents may enrich the extract with phenolic antioxidants, non-polar solvents might be better suited for isolating bioactive compounds with direct cytotoxicity. Overall, the variation in total phenolic and flavonoid contents due to extraction polarity significantly influenced both the antioxidant and cytotoxic properties of *U. dioica*.

### General evaluation

The present study underscores the critical influence of extraction solvent polarity on the chemical composition and multifaceted biological activities of *U. dioica*. The polar ME exhibited a significantly higher total phenolic and flavonoid content, aligning with its superior antioxidant activity demonstrated by DPPH and CUPRAC assays. Conversely, despite its lower phenolic content, the non-polar HE exhibited stronger cytotoxicity across A549, MDA-MB-231, and HCT116 cell lines, indicating the role of lipophilic cytotoxic constituents in the anticancer potential of *U. dioica*. Additionally, both extracts induced apoptosis and G0/G1 cell cycle arrest, with HE showing a more pronounced effect, suggesting that these extracts may exert their antiproliferative activities through apoptosis induction and modulation of cell cycle checkpoints.

In terms of antimicrobial activity, the HE extract demonstrated measurable inhibitory effects against a wider range of microorganisms, including both Gram-positive and Gram-negative bacteria as well as fungi, compared to ME; however, the inhibition zones were modest in size and substantially smaller than those produced by standard antibiotics. These findings collectively suggest that *U. dioica* holds promise as a bioactive plant with antioxidant, cytotoxic, and antimicrobial potential, while also highlighting that extraction methods significantly influence its total phenolic and flavonoid contents and observed biological effects. Future studies should prioritize bioactivity-guided fractionation and mechanistic analyses to isolate the specific compounds responsible for the pharmacological effects of *U. dioica* and to clarify the molecular pathways involved.

## Conclusion

The findings of this study suggest that *U. dioica* is a promising natural source of biologically active compounds with antioxidant, cytotoxic, and antimicrobial potential. The methanolic extract exhibited strong antioxidant activity due to its high phenolic and flavonoid content, while the hexane extract showed more potent cytotoxic and pro-apoptotic effects across multiple cancer cell lines, despite its lower antioxidant capacity—likely reflecting the presence of lipophilic bioactive constituents. Both extracts also induced apoptosis and G0/G1 phase arrest, suggesting a capacity to affect cancer cell proliferation through distinct cellular mechanisms. The modest antimicrobial activity observed, particularly with the hexane extract, further supports the potential application of *U. dioica* as a complementary agent in managing microbial infections. Importantly, the outcomes of this study emphasize the need for further research, including bioactivity-guided fractionation, in vivo validation, and exploration of synergistic effects with conventional therapies, to better understand and harness the pharmacological potential of *U. dioica*. Future studies examining the expression of key regulatory genes involved in cell cycle control (e.g., p53, p21, cyclins, CDKs) in response to *U. dioica* extracts will provide valuable insights into its underlying mechanisms of action. These findings also underscore the critical role of solvent polarity in determining the total phenolic and flavonoid contents and biological activities of *U. dioica* extracts. Acidified methanol effectively extracted phenolic compounds with antioxidant properties, whereas hexane enriched the extracts with lipophilic compounds exhibiting cytotoxic and antimicrobial effects, such as fatty acids and triterpenoids. This compound–activity relationship highlights the value of strategic solvent selection when targeting specific biological outcomes in plant-based extract research.

## Supplementary Information

Supplementary Material 1.

Supplementary Material 2.

Supplementary Material 3.

## Data Availability

The datasets used and/or analysed during the current study are available from the corresponding author on reasonable request.

## Abbreviations

A549: Human lung cancer
BEAS-2B: Human healthy lung cell
CE: Catechin equivalent
CUPRAC: Cupric Ion Reducing Antioxidant Capacity
DMSO: Dimethyl sulfoxide
DPPH: 2,2-Diphenyl-1-picrylhydrazyl
ELISA: Enzyme Linked Immunostaining Assay
GAE: Gallic acid equivalent
HCT116: Human colon cancer
HE: Hexane extract
MDA-MB-231: Human breast cancer
ME: Methanolic extract
MTT: 3-(4,5-Dimethylthiazol-2-yl)-2,5-diphenyltetrazolium bromide
PBS: Phosphate-buffered saline
RIPA: Radio-Immunoprecipitation assay
TE: Trolox equivalent
TFC: Total flavonoid content
TPC: Total phenolic content
