# Supplementary figures and images for: Comparative chemical and biological evaluation of Urtica dioica extracts obtained by methanol and hexane: antioxidant, cytotoxic, apoptotic, and antimicrobial potentials
Source: BMC Complement Med Ther. 2025 Dec 7;26:13. doi: 10.1186/s12906-025-05211-3 (PMC12797621; doi:10.1186/s12906-025-05211-3)

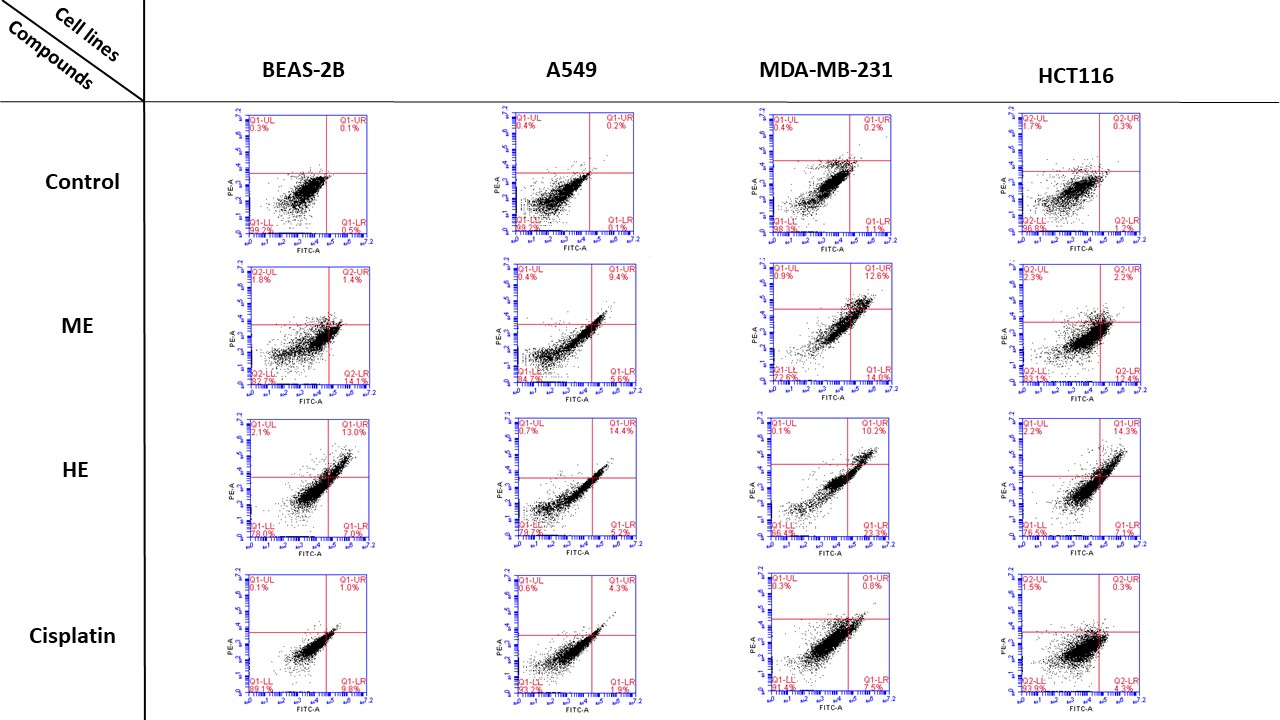

Supplement: Supplementary file 1 — Supplementary Material 1. [file 12906_2025_5211_MOESM1_ESM.jpg]

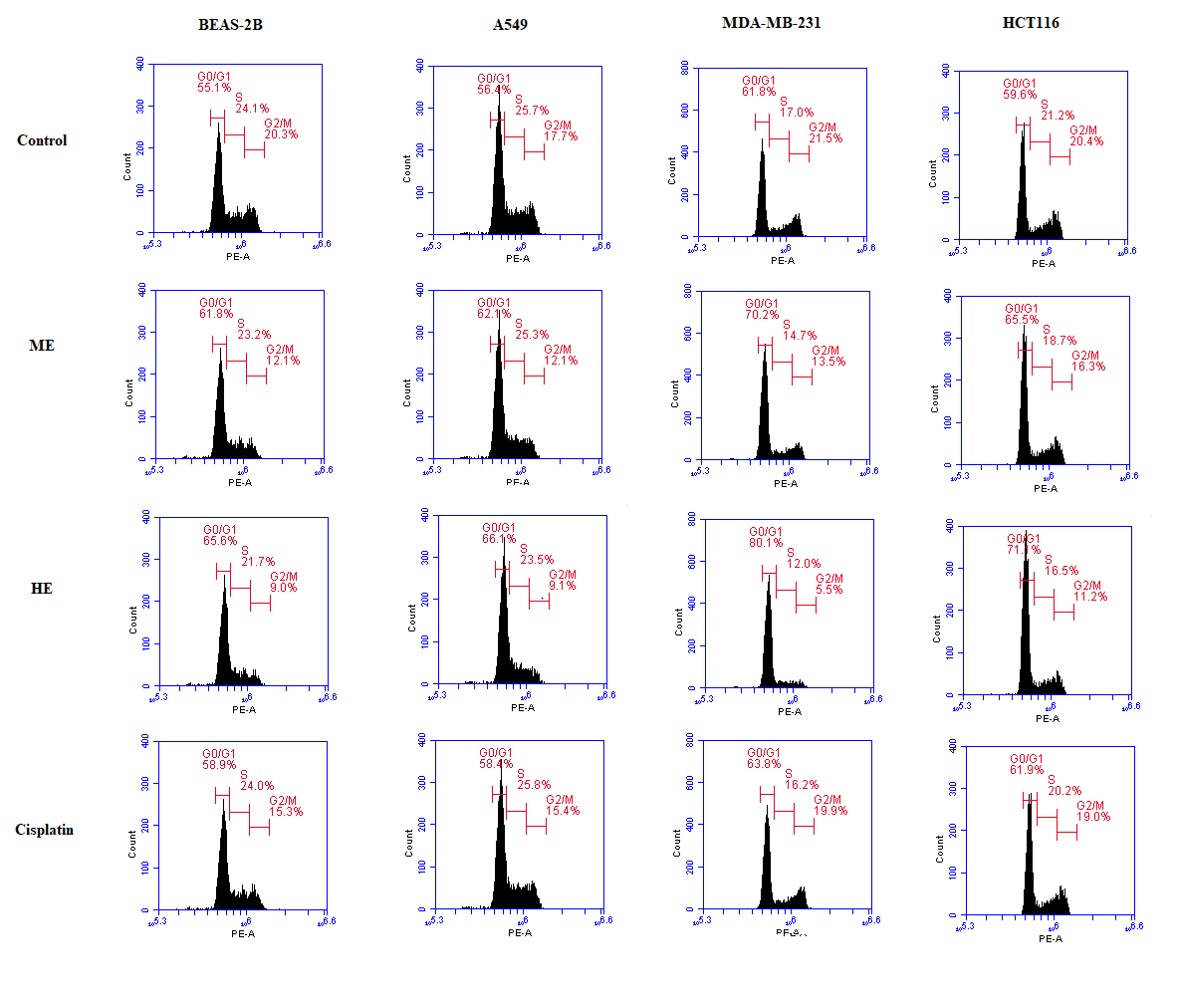

Supplement: Supplementary file 2 — Supplementary Material 2. [file 12906_2025_5211_MOESM2_ESM.png]
