# Supplementary material for: Comparative chemical and biological evaluation of Urtica dioica extracts obtained by methanol and hexane: antioxidant, cytotoxic, apoptotic, and antimicrobial potentials
Source: BMC Complement Med Ther. 2025 Dec 7;26:13. doi: 10.1186/s12906-025-05211-3 (PMC12797621; doi:10.1186/s12906-025-05211-3)
Supplement: Supplementary file 3 — Supplementary Material 3. [file 12906_2025_5211_MOESM3_ESM.pdf]

Supplemental information for IC50 Calculation

ME

BEAS-2B

Conc. (µg/ml)	% Viability
0,78	70,42038217
1,56	63,29299363
3,12	55,91082803
6,25	42,27388535
12,5	20,89171975
25	10,76433121
50	3,949044586
100	1,076433121
200	-5,73248408

IC50 = 5,06

A549

Conc. (µg/ml)	% Viability
0,78	90,0955414
1,56	82,52866242
3,12	72,99363057
6,25	58,45859873
12,5	41,5477707
25	29,1656051
50	10,89171975
100	1,146496815
200	0,50955414

8,02

MDA-MB-231

Conc. (µg/ml)	% Viability
0,78	73,48387097
1,56	68,16129032
3,12	54,03225806
6,25	39,83870968
12,5	27,48387097
25	19,61290323
50	5,967741935
100	0,709677419
200	-5,64516129

3,56

HCT116

Conc. (µg/ml)	% Viability
0,78	81,22929936
1,56	70,66242038
3,12	65,94904459
6,25	60,05732484
12,5	47,89808917
25	30,70063694
50	18,66242038
100	2,191082803
200	1,993630573

7,45

HE

BEAS-2B

Conc. (µg/ml)	% Viability
0,78	75,77498504
1,56	69,12627169
3,12	53,12986236
6,25	38,58168761
12,5	31,46618791
25	18,36205865
50	14,67145422
100	2,497905446
200	1,856971873

IC50 = 4,11

A549

Conc. (µg/ml)	% Viability
0,78	87,92296441
1,56	70,64602633
3,12	52,48171624
6,25	38,16821063
12,5	23,12042906
25	12,92540224
50	2,04778157
100	1,560214529
200	-6,92345197

4,17

MDA-MB-231

Conc. (µg/ml)	% Viability
0,78	78,33241909
1,56	56,23186561
3,12	48,85957213
6,25	36,34777839
12,5	27,75644542
25	19,46900713
50	4,701042238
100	2,970927043
200	0,86615469

3,12

HCT116

Conc. (µg/ml)	% Viability
0,78	79,49439298
1,56	62,62457338
3,12	59,03949293
6,25	43,09117504
12,5	18,21550463
25	15,48512921
50	7,609458801
100	1,271087275
200	0,854217455

3,89

CISPLATIN

BEAS-2B

Conc. (µg/ml)	% Viability
0,78	100,5321508
1,56	90,11086475
3,12	87,6940133
6,25	76,43015521
12,5	64,01330377
25	50,84257206
50	42,83813747
100	30,28159645
200	15,45454545

IC50 = 27,09

A549

Conc. (µg/ml)	% Viability
0,78	98,77674163
1,56	95,9243006
3,12	93,83982447
6,25	83,40098738
12,5	72,52331322
25	62,30389468
50	51,31651125
100	41,97476687
200	32,75534833

60,28

MDA-MB-231

Conc. (µg/ml)	% Viability
0,78	102,195122
1,56	98,8691796
3,12	86,87361419
6,25	76,20842572
12,5	63,99113082
25	51,99556541
50	40,66518847
100	32,2172949
200	22,66075388

31,22

HCT116

Conc. (µg/ml)	% Viability
0,78	104,6077894
1,56	93,29127811
3,12	91,73011519
6,25	88,65057597
12,5	85,12342293
25	72,50685683
50	54,6922655
100	47,7893582
200	25,45255074

76,12
